# The Potential of Artificial Cells Functioning under *In Situ* Deep-Sea Conditions

**DOI:** 10.1021/acssynbio.4c00441

**Published:** 2024-10-01

**Authors:** Yutetsu Kuruma, Hidetaka Nomaki, Noriyuki Isobe, Daisuke Matsuoka, Yasuhiro Shimane

**Affiliations:** † Institute for Extra-cutting-edge Science and Technology Avant-garde Research (X-star), Japan Agency for Marine-Earth Science and Technology (JAMSTEC), 2-15 Natsushima-cho, Yokosuka, Kanagawa 237-0061, Japan; ‡ Biogeochemistry Research Center, Research Institute for Marine Resources Utilization (MRU), Japan Agency for Marine-Earth Science and Technology (JAMSTEC), 2-15 Natsushima-Cho, Yokosuka, Kanagawa 237-0061, Japan; § Center for Earth Information Science and Technology (CEIST), Research Institute for Value-Added-Information Generation (VAiG), Japan Agency for Marine-Earth Science and Technology (JAMSTEC) 3173-25 Showa-machi, Kanazawa-ku, Yokohama, Kanagawa 236-0001 Japan

**Keywords:** Artificial Cells, Cell-Free Gene Expression, Origin of Life, Deep-Sea, Membrane Vesicles, In-Situ Experiment

## Abstract

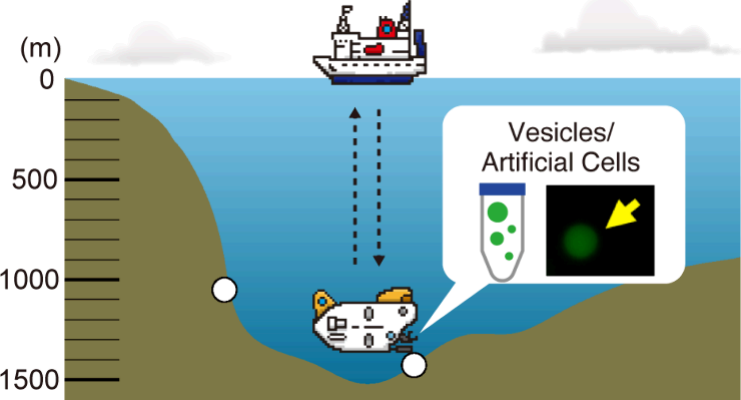

Artificial cells with reconstructed cellular functions could serve as practical protocell models for studying the early cellular life on the Earth. Investigating the viability of protocell models in extreme environments where life may have arisen is important for advancing origin-of-life research. Here, we tested the survivability of lipid membrane vesicles in deep-sea environments. The vesicles were submerged in the deep-sea floor with a human-occupied vehicle. Although most of the vesicles were broken, some vesicles maintained a spherical shape after the dives. When a cell-free protein synthesis system was encapsulated inside, a few vesicles remained even after a 1,390 m depth dive. Interestingly, such artificial cells could subsequently synthesize protein in a nutrient-rich buffer solution. Together with on shore experiments showing artificial cells synthesized protein under high pressure, our results suggest artificial cells may be able to express genes in deep-sea environments where thermal energy is available from hydrothermal vents.

## Introduction

The question of how the first organisms emerged and survived in the early Earth environment is an open question attracting not only scientists but also general interest.^1^ It is widely accepted that life originated in what we now call extreme environments, for example, hot spring geysers or deep-sea hydrothermal vents.^2−4^ However, there are limitations in the experimental approach to answering these questions because the extremophiles living in such extreme environments have already been adopted after billions of years of evolution.^5^ Protocells that have simple components (i.e., RNA and fatty acid membrane) and a self-replicable feature are thought to have existed before life. How primitive cells that could have existed during the evolution of the protocells into LUCA (Last Universal Common Ancestor) survived in such an environment is enigmatic. The most direct approach to answering this question would be to place pseudocells, which model the minimal properties of cells, in an environment and observe how they behave.

The attempts to build up living cells or cell mimicries by assembling biomolecules such as proteins, lipids, and DNAs are expanding the field of life science, and such man-made cells are called artificial cells or synthetic cells.^6^ Spherical membrane vesicles made of defined phospholipids are important materials as a chassis for artificial cells since they can encapsulate molecules and conduct the activation of cell functions inside.^7^ For instance, by encapsulating all necessary enzymes and small molecules for transcription and translation, we can perform gene expression inside the membrane vesicles.^8−10^ Although most artificial cell research is carried out in biological laboratories, how well they can function in a real environment has not been studied so far. In the context of harnessing artificial cells for the origin of life studies, delivering the artificial cells into the extreme environment that simulates the potential birthplace of life and analyzing their consequences are important. Furthermore, understanding the stability of membrane vesicles under high-pressure and low-temperature environments will open possibilities for applications in materials engineering.

In this study, we tested the viability of giant unilamellar vesicles (GUVs) and artificial cells in deep-sea environments during three dives of human-occupied vehicle (HOV) *Shinkai 6500*. We found that GUVs were much stressed by seawater and deep-sea conditions (i.e., high salinity and high pressure, respectively), but, even so, a small number of vesicles survived when the osmolality inside was well adjusted. We also performed high-pressure experiments on land to see whether artificial cells could express protein. These efforts may open up new aspects of the origin of life study and artificial cell research, and provide an opportunity to consider models of the emergence of cellular life under realistic conditions.

## Results and Discussion

### Set up for Deep-Sea Experiments

**Figure 1:**
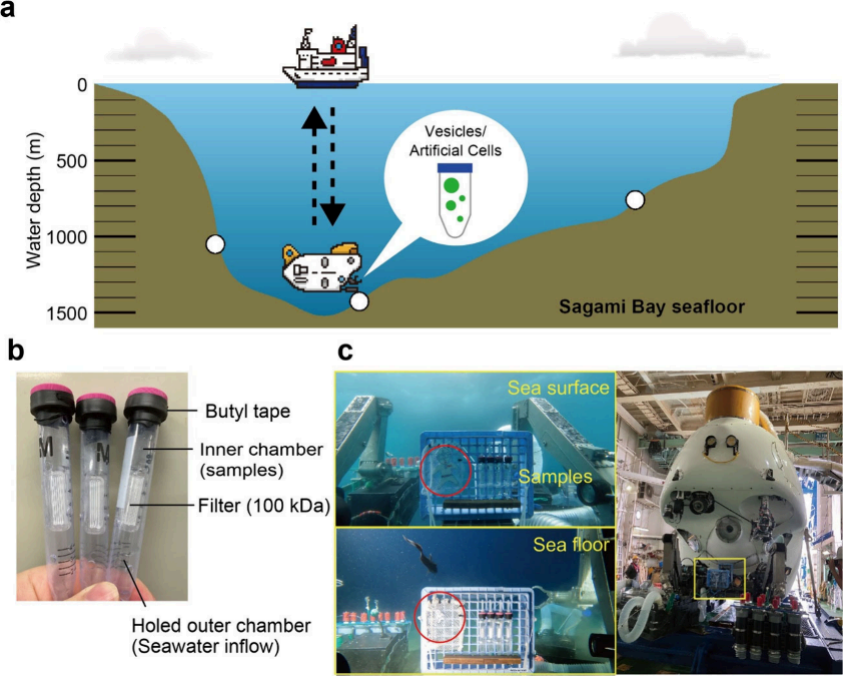
Delivery of GUVs or artificial cells to deep-sea by the submersible. (a) Schematic view (East–West bathymetric dimension) of Sagami Bay, Japan, where the submersible dives were performed. We had three dives at the different water depths (760, 1,050, and 1,390 m, indicated as white circles) of seafloor with the support of the research vessel *Yokosuka*. (b) The sample-containing ultrafiltration units delivered to the deep-sea. (c) *Shinkai 6500* installing the test samples. Red circles are a Styrofoam model “Yutetsu-Kun” indicating the change in water pressure (see also Movie S1).

To investigate the viability of membrane vesicles, three deep-sea floor locations were selected from the bathyal depths (760, 1,050, and 1,390 m) of Sagami Bay, Japan (Figure 1a). The membrane vesicles prepared with phospholipids were mixed with seawater and placed in the inner chamber of an ultrafiltration unit, Ultracon. The inner chamber was set to the unit, and the cap was sealed tightly with butyl tape to avoid the inflow of surrounding seawater, along with avoiding the outflow of GUVs or artificial cells to environments. The outer chamber was holed to allow surrounding seawater inflow to compensate for the hydrostatic pressure (Figure 1b). Because there is water exchange between the filter membrane of Ultracon (Figure S1), the effects of the deep-sea pressure and temperature influence vesicles during the dive (Figure S2). Prepared tubes were attached to the sample basket of the HOV *Shinkai 6500*, with a humanoid model made of Styrofoam “Yutetsu-Kun” that was used as an indicator of high pressure (Figure 1c and Movie S1).

### Vesicle Viability after the Deep-Sea Dives

The first hurdle for the viability of GUVs is to survive osmotic stress when mixed with seawater. The osmolality inside the vesicles must be equal to or lower than that in seawater. Otherwise, the influx of water causes the vesicles to burst. Izumi et al. reported that the osmolality of seawater in the Miura coast, which is facing Sagami Bay, is 1000 mmol/kg.^11^ We first tested the osmolality of different concentrations of sucrose (500, 750, and 1000 mM) in a Tris buffer. The results showed that the correlation between sucrose concentration and mmol/kg was 1.484 (Figure S3).

**Figure 2:**
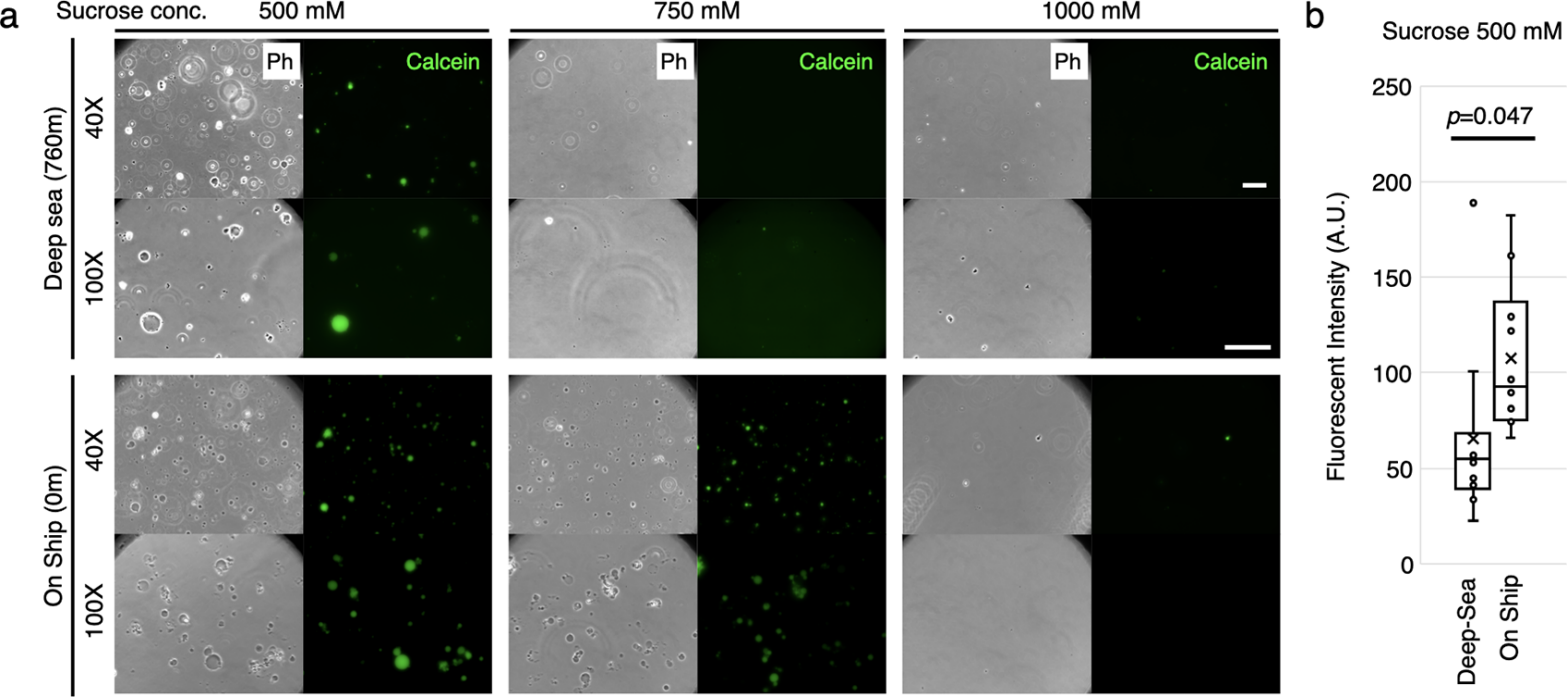
GUVs submerged into 760 m water depth at Sagami Bay deep-sea floor. (a) The GUVs encapsulating 500 mM, 750 mM, or 1000 mM sucrose were prepared with POPC 100% mol and delivered into 760 m depth of deep-sea or remained on the ship. The resulting GUVs were observed with 40× and 100× objective lenses in the set of a phase contrast set (Ph) or fluorescent for the encapsulated calcein. Scale bar: 40 μm. (b) Statistical analysis of fluorescent intensities of calcein inside the 500 mM sucrose-encapsulating vesicles is shown in the comparison of dived (Deep Sea) and non-dived (On Ship) samples. For each sample, 10 vesicles were analyzed.

We prepared GUVs encapsulating calcein and 500 mM, 750 mM, or 1000 mM sucrose and delivered them to 760 m water depth of deep-sea floor for 4.5 h (Figure S2). As a control without deep-sea exposure, we stored the same GUV samples on the ship at 4 °C, which is the same as the *in situ* water temperature. When 500 mM sucrose was encapsulated (osmolality ca. 700 mmol/kg), some GUVs were found to have retained their spherical shape (Figure 2a) but we could not find GUVs when encapsulating 750 mM and 1000 mM sucrose. On the other hand, the control GUVs stored on the ship retained spherical shape in both 500 and 750 mM sucrose-encapsulating GUVs. Since the fluorescence of the encapsulated calcein was observed, we assume that there is no significant leakage from the GUVs having 700 mmol/kg osmotic pressure, even during deep-sea dive. We also observed that the structure of the vesicle surface changed to rough, attaching small vesicles or oils. This may be due to the effect of seawater since both deep-sea and on-ship samples show similar structural changes. A 1000 mM sucrose in GUVs has too high osmolality (ca. 1500 mmol/kg); therefore, they were ruptured by osmolality shock by seawater. Using the GUVs prepared with 500 mM sucrose, we performed statistical analysis to compare the fluorescence intensities of the internal calcein between dived and not-dived samples. However, there was no significant difference between them (Figure 2b). This means that high pressure in the deep-sea environment does not affect the leakage of calcein.

**Figure 3:**
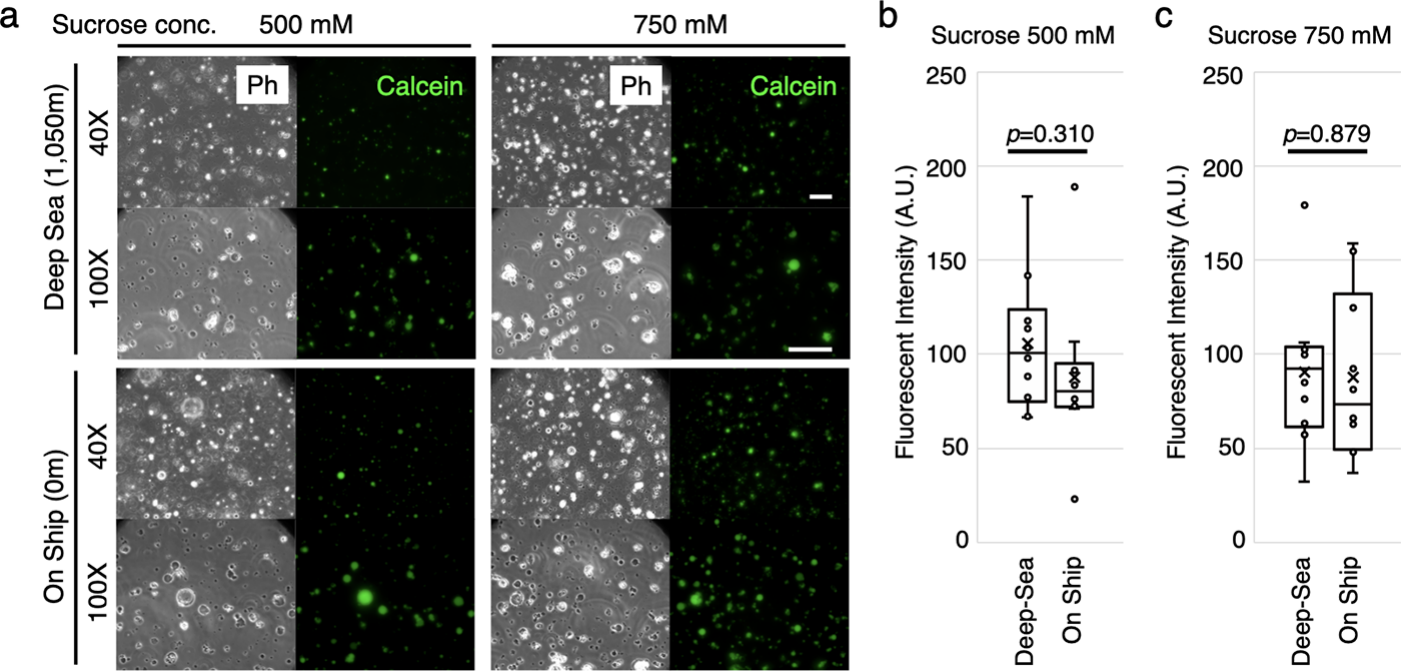
GUVs submerged into 1,050 m water depth. The GUVs encapsulating calcein and 500 mM or 750 mM sucrose were prepared with 100% mol POPC. The GUVs were delivered into 1,050 m depth of deep-sea or remained on the ship. (a) The resulting GUVs were observed with 40× and 100× objective lenses in the set of a phase contrast set (Ph) or fluorescent for the encapsulated calcein. Scale bar: 40 μm. Statistical analysis of the fluorescent intensities of calcein inside the (b) 500 mM or (c) 750 mM sucrose-encapsulating vesicles are shown in the comparison of dived (Deep Sea) or non-dived (On Ship) samples. For each sample, 10 vesicles were analyzed.

Next, we tested the GUVs viability in a deeper environment; at 1,050 m water depth (3.2 °C) for 5 h (Figure S2). The GUVs were prepared with 500 mM and 750 mM sucrose inside. Different from the first experiment, this time we observed the retention of spherical shape and calcein fluorescence in the GUVs containing both 500 and 750 mM sucrose even after the 1,050 m dive (Figure 3a). We observed that there was no statistical difference in the fluorescent intensities between the dived and non-dived GUVs in both sucrose concentrations (Figure 3b and c), suggesting that GUV membrane does not tolerate calcein leakage even in the 1,050 m deep-sea environment.

### Deep-Sea Experiment with Artificial Cells

**Figure 4:**
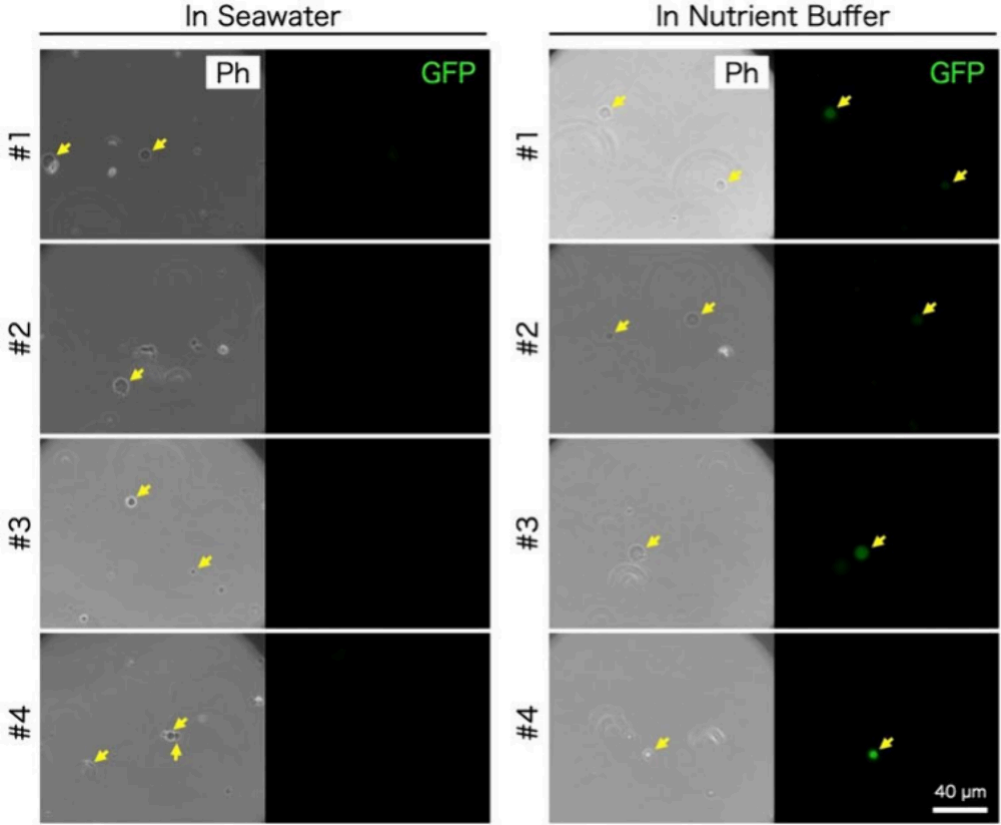
Artificial cells submerged into 1,390 m water depth. The artificial cells encapsulating a cell-free system (PURE system) and 200 mM sucrose were prepared with POPC 100% mol. The cells were delivered into a 1,390 m depth of deep-sea. The resulting GUVs were concentrated and resuspended within seawater or a nutrient buffer. After the incubation at 37 °C for hours, the cells were observed with 100× objective lenses in the set of a phase contrast set (Ph) or fluorescent for the synthesized GFP. Scale bar: 40 μm.

For the third deep-sea experiment, we prepared artificial cells by encapsulating a reconstructed cell-free protein synthesis system, the PURE system, with the DNA of green fluorescent protein (GFP) inside 100% POPC GUVs. A 200 mM or 500 mM sucrose was encapsulated together with the PURE system. In the case of 500 mM sucrose used, however, all artificial cells disappeared after being mixed with seawater. This is perhaps because of the high osmolality of the inner mixture compared with the outer seawater since the PURE system itself already has high osmolality (ca. 900 mmol/kg). Contrarily, a small portion of artificial cells remained spherical with the 200 mM sucrose encapsulating artificial cells even after mixing with seawater (Figure S4). The artificial cells containing 200 mM sucrose were submerged in the 1,390 m depth of deep-sea (2.5 °C) for about 5 h (Figure S2). The recovered cells were stored at 4 °C until return to the laboratory; then 4 days later, the artificial cells were concentrated by centrifugation within seawater and incubated at 37 °C for a few hours. However, we could not find any fluorescence from the inside of artificial cells. Next, we resuspended the concentrated artificial cells in the nutrient-rich buffer equivalent to the PURE system buffer, containing 20 amino acids but lacking NTPs and tRNAs. After a few hours of incubation, we found that several artificial cells exhibited green fluorescence (Figure 4). Although most of the artificial cells were inactive, visually ca. 10% of the artificial cells that survived from deep-sea showed fluorescence. We do not know the exact reason why the other 90% of the survived artificial cells do not synthesize GFP. We speculate that some of the PURE system components leaked out irreversibly or some of the seawater components seeped into the artificial cells and inhibited protein synthesis.

These results show that the transcriptional and translational machinery of the PURE system can remain within the GUVs even after exposure to a deep-sea environment. However, the small compounds, such as amino acids or ions, that are also needed for protein synthesis may have leaked outside of GUV due to mixing with seawater or diving into the deep sea, resulting in no protein synthesis occurring under the seawater condition. In turn, the leaked components could influx into the GUVs when resuspended in nutrient buffer.

Although there are many cell-free systems, we chose the PURE system because all of the components and those concentrations are defined. This fact is beneficial when analyzing encapsulated mixture in detail without any black box: for example, it is technically possible to identify what molecules leaked from the vesicles and reveal why protein synthesis was not achieved in seawater.

### Effect of High Pressure on Gene Expression in Artificial Cells

**Figure 5:**
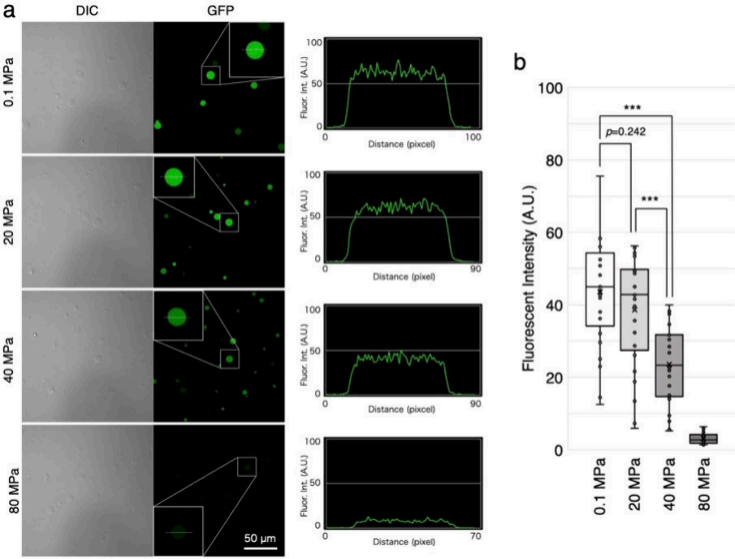
On shore high-pressure experiments of artificial cells. The artificial cells were incubated under high pressure of 20, 40, and 80 MPa. As a control, the same reaction was performed also at atmospheric pressure (0.1 MPa). (a) The resulting cells were observed by confocal microscopy with the set of Differential Interference Contrast (DIC) and 488 nm laser (GFP). Typical cells shown in the insets were analyzed to show the fluorescent intensity by plot profile using ImageJ. Bar: 50 μm. (b) 25 cells at each pressure were measured, and the fluorescence intensity of the artificial cells was compared. ***: 0.0005.

To simulate artificial cell experiments around deep-sea hydrothermal vents, we tested if artificial cells can synthesize protein under a high pressure on land. The artificial cells were exposed to the pressure of 20, 40, or 80 MPa while being heated at 37 °C. After 3 h, the resulting artificial cells were observed by confocal microscopy. The results showed that the artificial cells exhibited the fluorescence of the synthesized GFP even being exposed to 20 MPa pressure (Figure 5a). This was similar to the control experiment performed under an atmospheric pressure (0.1 MPa). However, the fluorescence intensity reduced at 40 MPa, and almost no fluorescence was observed when the cells were exposed to 80 MPa pressure. Statistical analysis comparing the fluorescence intensity under each pressure showed that there was no decrease at a pressure of 20 MPa, but the fluorescence intensity decreased to 50% at a pressure of 40 MPa (Figure 5b). These results are consistent with a previous report showing that *E. coli* can survive up to a pressure of 50 MPa.^12^ In general, hydrothermal vents are located at a depth of about 1,000 m deep-sea (10 MPa), so the artificial cells would be able to function near the hydrothermal vents.

## Conclusion

In this study, we submerged GUVs and artificial cells in deep-sea environments during three submersible dives on the deep-sea floor (Table S1). We found two challenges that will have to be overcome in the realization of artificial cells functioning in the deep-sea hydrothermal vent area.

The first challenge is the stability of the GUV in seawater. We found that GUVs containing 1000 mM sucrose disappeared after mixing with seawater (Figure 2a). Additionally, artificial cells encapsulating the PURE system with 500 mM sucrose also disappeared. These facts suggest that the GUVs were ruptured by hypoosmotic pressure just after being mixed in seawater. It could be possible to minimize the osmotic stress by perfectly regulating the osmotic pressure inside a GUV. Moreover, implementing mechanosensitive membrane channels might reduce the risk of rupture by releasing the influx of water to the outside. According to Izumi et al., the presence of DSPE-PEG5000 on the GUV membrane blocks ion access which causes vesicle distraction.^11^ Encapsulating hydrogel in GUV or implementing cytoskeleton on the GUV membrane may increase the stability of a spherical GUV.

Another challenge is the leakage of small molecules from the GUV lumen to the outside. Since we observed protein synthesis inside artificial cells that traveled to 1,390 m water depth of the deep-sea, it is sure that transcriptional and translational machinery are maintained in the survived GUVs. The nutrient-rich buffer allowed protein synthesis inside GUVs, whereas seawater did not. About this issue, we speculate that small-size molecules, such as amino acids or Mg^2+^ ions, were not maintained in sufficient concentrations for protein synthesis inside GUV during dilution in seawater and/or exposure to deep-sea environments. The leaked molecules could passively diffuse into the GUV when we put the artificial cells in the nutrient-rich buffer; thus, protein synthesis occurred. To overcome this problem, we may need to form a tight lipid membrane through modification of the lipid composition. The use of cholesterol^8^ or isoprenoid-type lipids may reduce the leak. Protein synthesis under high pressure is feasible up to 40 MPa (Figure 5).

Our experiment is the first to be conducted using artificial cells in a deep-sea environment, which is thought to be the birthplace of life on Earth. The results of this study suggest that artificial cells constructed from minimal factors may be able to survive in the deep-sea environment. It has been considered that the first cell form was rather simpler than modern cells before evolutional games. Likewise, the first man-made cells could be the simplest cells that can be barely alive under the defined conditions of a laboratory. The cells that existed long ago and the cells that will be born in the future are quite the opposite on the timeline, but curiously, these cells possess very similar features. We believe that experimentally investigating how these cells fit into the environment and how to maintain their vital functions is an important new aspect in the origin of life study and a new challenge in synthetic biology.

## Methods

### Materials

1-Palmitoyl-2-oleoyl-glycerol-3-phosphocholine (POPC) was purchased from Avanti Polar. The PURE system (PURE*frex*2.0) was purchased from GeneFrontier company (Japan). Ultrafiltration unit (Ultracon-100 kDa) was purchased from Merck Millipore.

### Vesicle Preparation

GUVs and artificial cells were prepared as s described previously.^13^ Briefly, 100 μL of 100 mM phospholipid solution dissolved in chloroform was transferred into a glass tube and evaporated by a gentle nitrogen gas flow with a processing vortex. The resulting lipid film was placed in a desiccator under low pressure overnight to completely remove the solvent. The dried lipid film was mixed with 500 mL of mineral oil and dissolved well by heating at 70 °C and mixing by a vortex to make a lipid-oil. Then 20 μL of 50 mM Tris-HCl (pH 7.6), containing 1 μM calcein and sucrose as indicated concentration, or reaction mixture of the PURE system, prepared as per manufacturer’s instruction, was added into the lipid-oil and mixed well by a vortex. So emulsified solution was layered on top of the outer solution composed of 50 mM Tris-HCl containing the same concentration of glucose as in the inner solution. In the case of artificial cells, the PURE system buffer including 200 or 500 mM glucose but lacking NTPs and tRNA mix was used as the outer solution. The resulting tube was centrifuged for 10 min at 15,000*g* at room temperature to form GUVs into the outer solution. After the removal of the upper oil phase by pipetting, the formed GUVs or artificial cells were collected from the bottom of the tube. The GUV solution generally became 30 μL.

### Ultrafiltration Unit

We employed a 100 kDa cut Ultracel-100 membrane 4 mL (Millipore) to exchange GUV-resuspending solution and seawater in deep-sea. To allow the influx of seawater to the membrane of the inner chamber, we made several holes with 5 mm (dia.) at the outer shell (Figure 1b). GUVs or artificial cells prepared as above were diluted with 5 mL of seawater, which was passed a 0.2 μm filter in advance, and transferred into the inner chamber of Ultracon and messed up with seawater without remaining air. To avoid the influx of seawater from the cap of the unit, we sealed the cap part tightly with butyl tape. The sample-installed units were fixed on the sample basket of the HOV *Shinkai 6500* with cable ties as shown in Figure 1c.

### GUVs Observation

1 mL of GUVs diluted in seawater was centrifuged for 10 min at 15,000*g* at room temperature and resuspended in 20 μL of seawater or the PURE system outer solution. The GUV samples thus concentrated were dropped onto a slide glass (Matsunami, 24 × 32 mm^2^) and covered with another slide glass sandwiching silicone grease used as a spacer, and observed by a fluorescence microscopy (Olympus IX73) implemented with 40× and 100× objective lenses. The GUVs were observed also by the phase contrast mode. The images were taken by an Andor Zyla 5.5 sCMOS camera with the software MetaMorph.

### Osmolality Measurement

The osmolality of solutions was measured by a vacuum pressure osmometer (WESCOR, U.S.) following the manufacturer’s instruction. The measurements were repeated three times, and their average was used for the study.

### High-Pressure Experiments

To verify there are material exchanges between the ultrafiltration membrane of the inner chamber, we demonstrate a high-pressure experiment as follows. The inner chamber filled with Milli-Q water was set to the Ultracon unit where the outer chamber was holed. The tubes were dipped into a Coomassie Brilliant Blue (CBB) solution within a plastic bag and set inside a pressure vessel. The pressure inside the vessel was increased up to 10 MPa, which is equivalent to 1,000 m depth of water, and kept at 2–4 °C for 5 h. After the Ultracon unit was collected, the color of the inner solution was observed.

To test the gene expression of artificial cells under high pressure, we demonstrated the following experiments. First, prepared artificial cells were housed in a plastic syringe where the needle side was capped (Figure S5). The syringes were packed in a plastic pack filled with water and then put inside pressure vessels. The temperature was kept at 37 °C by a water bath incubator for 3 h. After the incubation under high pressure, the artificial cells were collected and observed by confocal microscopy (Nikon A1R system).

## Supporting Information Available

Water exchange between the ultrafiltration membrane; record of water depth, temperature, and salinity; osmotic pressure of sucrose solutions; artificial cells diluted in seawater; set up for high-pressure artificial cell experiments; summary of submerged samples (PDF)
Digest movie of deep-sea diving at water depth of 1,390 m (MP4)
