# Supplementary material for: The Potential of Artificial Cells Functioning under In Situ Deep-Sea Conditions
Source: ACS Synth Biol. 2024 Oct 1;13(10):3144–9. doi: 10.1021/acssynbio.4c00441 (PMC11494692; doi:10.1021/acssynbio.4c00441)
Supplement: Supplementary file 1 — sb4c00441_si_001.pdf [file sb4c00441_si_001.pdf]

Supplementary Information

The potential of artificial cells functioning under *in situ* deep-sea conditions

Yutetsu Kuruma[1*], Hidetaka Nomaki[1], Noriyuki Isobe[2], Daisuke Matsuoka[3], Yasuhiro Shimane[1]

[1]Institute for Extra-cutting-edge Science and Technology Avant-garde Research (X-star), Japan Agency for Marine-Earth Science and Technology (JAMSTEC), 2-15 Natsushima-cho, Yokosuka, Kanagawa 237-0061, Japan
[2]Biogeochemistry Research Center, Research Institute for Marine Resources Utilization (MRU), Japan Agency for Marine-Earth Science and Technology (JAMSTEC), 2-15 Natsushima-Cho, Yokosuka, Kanagawa, 237-0061, Japan
[3]Center for Earth Information Science and Technology (CEIST), Research Institute for Value-Added-Information Generation (VAiG), Japan Agency for Marine-Earth Science and Technology (JAMSTEC) 3173-25 Showa-machi, Kanazawa-ku, Yokohama, Kanagawa 236-0001 Japan
* Corresponding Author: E-mail address: ykuruma@jamstec.go.jp (Yutetsu Kuruma)

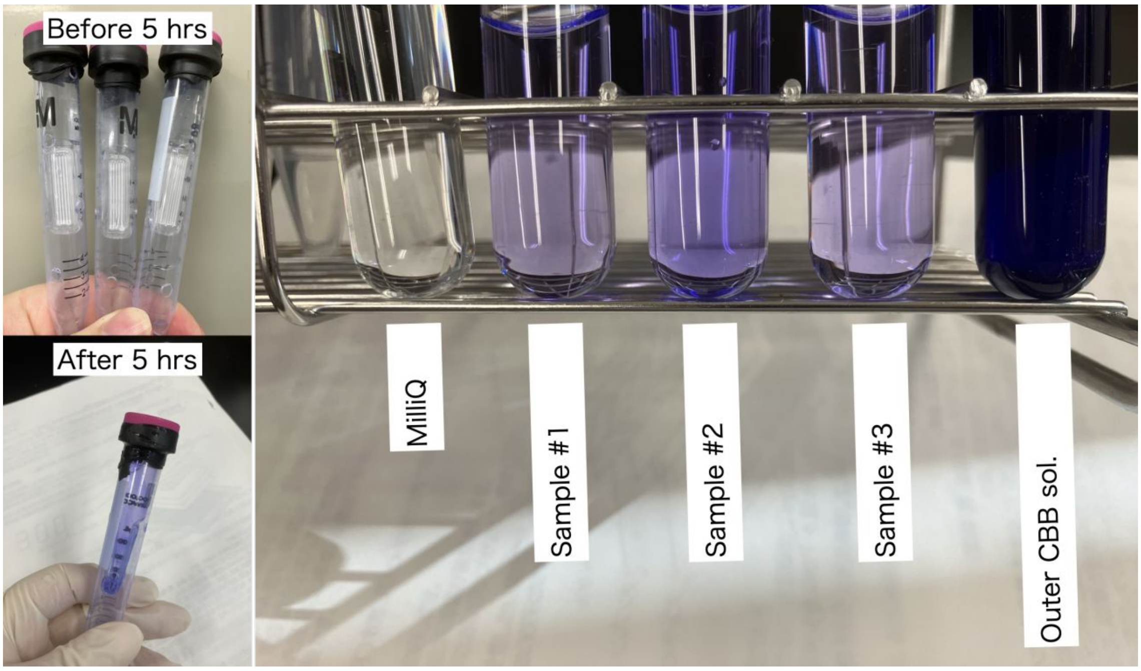

Figure S1. Water exchange between the ultrafiltration membrane. The inner chamber filed with MilliQ water was set to the Ultracon unit where the outer chamber was holed. The tubes were dipped into a Coomassie Brilliant Blue (CBB) solution within a plastic bag and set inside a pressure vessel. The pressure inside the vessel was increased up to 10 MPa, which is equivalent to 1,000 m depth of water, and kept at 2-4 ˚C for 5 hours. After collecting the Ultracon unit, the color of the inner solution was observed.

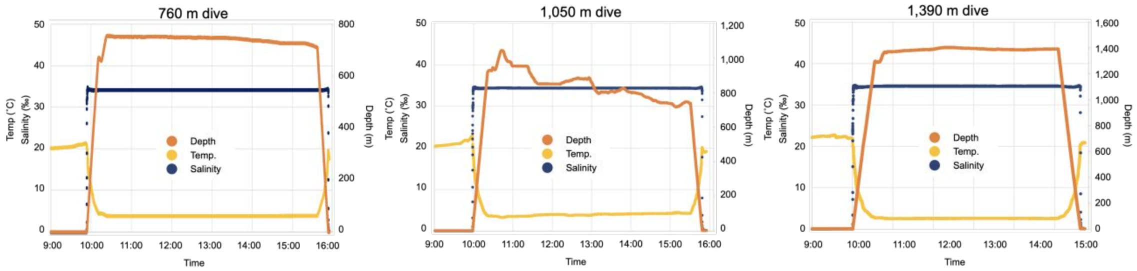

Figure S2. The water depth, temperature, and salinity during the dives of HOV *Shinkai6500* measured with CTDO sensor (Seabird SBE19).

Sucrose (mM)	Test 1	Test 2	Test 3	Average	S.D.
0	32	20	22	24.67	5.25
500	690	691	708	696.33	8.26
750	1065	1065	1068	1066.00	1.41
1000	1520	1551	1556	1542.33	15.92

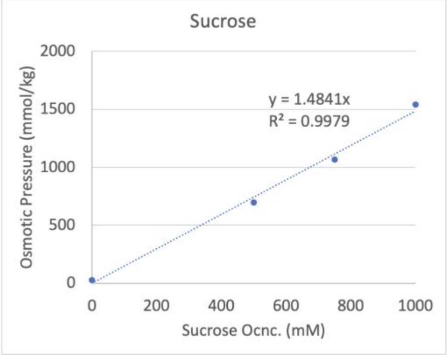

Figure S3. Osmotic pressure of sucrose solutions. The osmotic pressure of sucrose solutions were measured by VAPRO® (WESCOR, U.S.). Each measurement was repeated three times and their average values were used for making the graph with the values of standard deviation (S.D.).

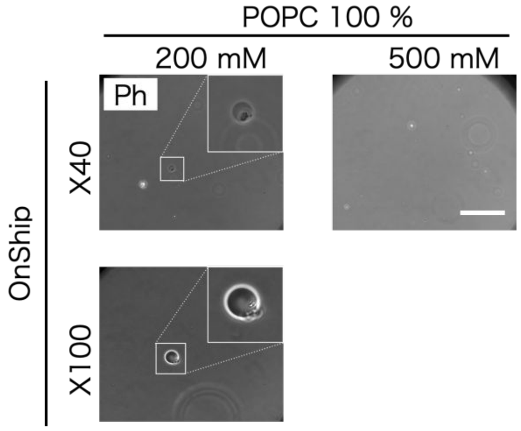

Figure S4. Artificial cells diluted in seawater. Artificial cells encapsulating a cell-free system (PURE system) and 200 mM or 500 mM sucrose were prepared with POPC 100 %mol. 20 μL of the cells were diluted in 5 mL seawater passed with a 0.22 μm filter. The resulting cells were observed with ×40 and ×100 objective lends in the set of a phase contrast set observation (Ph). Scale bar: 40 μm.

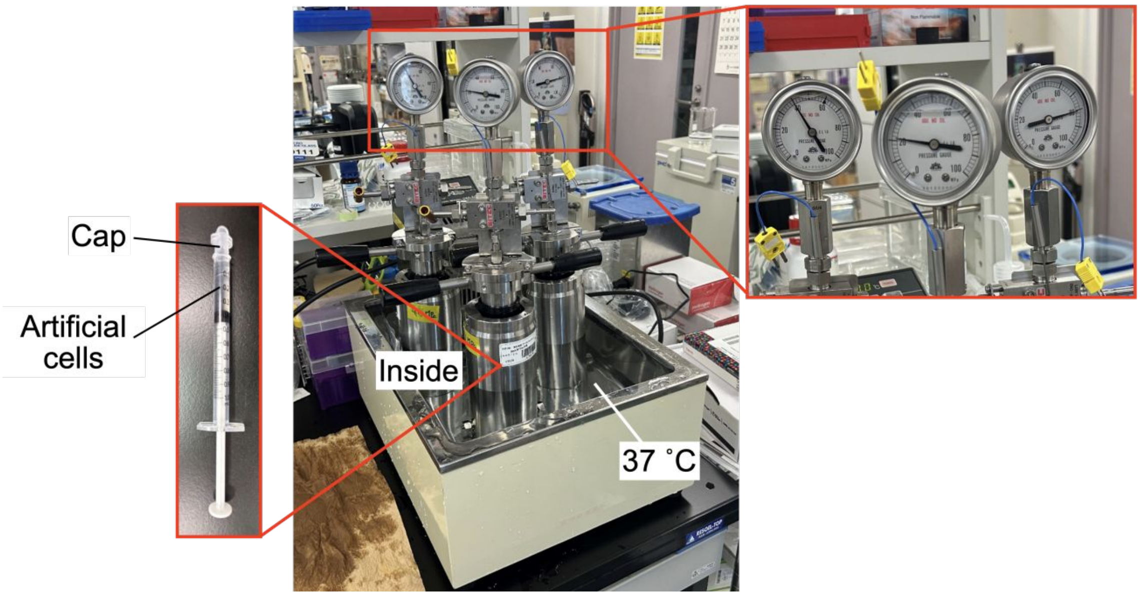

Figure S5. Set up for on shore high-pressure artificial cell experiments. The prepared artificial cells were housed in the syringe with a cap and packed inside a plastic bag with filling water. Three pressures (20, 40, and 80 MPa) and atmospheric pressure were tested. The temperature was kept at 37 ˚C to mimic the environment near hydrothermal vents.

Table S1. Summary of submerged samples.

#	Sample	Lipid (%mol)	Sucrose Conc. (mM)	Maximum water depth (m)	LAT, LNG
1	GUVs	POPC (100)	500, 750, 1000	760	35˚04.2N, 139˚32.4E
2	GUVs	POPC (100)	500, 750	1050	35˚01. 0N, 139˚13.3E
3	Artificial cells	POPC (100)	200, 500	1390	34˚59.7N, 139˚23.2E
